# Multiple pH Regime Molecular Dynamics Simulation for p*K* Calculations

**DOI:** 10.1371/journal.pone.0020116

**Published:** 2011-05-27

**Authors:** Lennart Nilsson, Andrey Karshikoff

**Affiliations:** 1 Department of Biosciences and Nutrition, Center for Biosciences, Karolinska Institutet, Huddinge, Sweden; 2 Institute of Molecular Biology, Bulgarian Academy of Sciences, Sofia, Bulgaria; University of Wales Bangor, United Kingdom

## Abstract

Ionisation equilibria in proteins are influenced by conformational flexibility, which can in principle be accounted for by molecular dynamics simulation. One problem in this method is the bias arising from the fixed protonation state during the simulation. Its effect is mostly exhibited when the ionisation behaviour of the titratable groups is extrapolated to pH regions where the predetermined protonation state of the protein may not be statistically relevant, leading to conformational sampling that is not representative of the true state. In this work we consider a simple approach which can essentially reduce this problem. Three molecular dynamics structure sets are generated, each with a different protonation state of the protein molecule expected to be relevant at three pH regions, and p*K* calculations from the three sets are combined to predict p*K* over the entire pH range of interest. This multiple pH molecular dynamics approach was tested on the GCN4 leucine zipper, a protein for which a full data set of experimental data is available. The p*K* values were predicted with a mean deviation from the experimental data of 0.29 pH units, and with a precision of 0.13 pH units, evaluated on the basis of equivalent sites in the dimeric GCN4 leucine zipper.

## Introduction

The p*K* values of the ionisable groups in proteins may differ significantly from those of free amino acids or model compounds. The main factors responsible for this effect, namely the charge-charge interactions and the desolvation penalty, are well understood [1]–[4]. The contribution of these factors to the p*K* shifts of the individual ionisable sites depends on their environment, hence an adequate description of the structural details of the protein molecule is of prime importance. The most common structural basis for p*K* calculation is the X-ray structure. It is known however that high resolution X-ray structure determination, especially on atomic and subatomic resolution, ends up with more than one conformation of some side chains. Also, in equilibrium proteins in solution populate a set of conformations, which can be significantly different from that observed in the crystalline state. In our earlier works these features were referred to as conformational heterogeneity [5]–[7].

Introduction of conformational flexibility in the p*K* calculations faces practical difficulties arising from the enormously large conformational space needed to be explored in order to select the statistically relevant protein conformers. Various approaches have been proposed to solve this problem. In order to reduce the computational size of the task Kieseritzky and Knapp [8] have introduced random conformational perturbations of groups participating in salt bridges. Although the approach is limited to the consideration of only part of the side chains, it addresses a very important problem, namely the possible overestimation of the contribution of the charge-charge interactions [9], [10]. In a series of works Alexov and Gunner [11]–[13], as well as Barth et al. [14] have developed and successfully used an approach for coupling of continuum electrostatic p*K* calculations with conformational sampling of side chain rotamers. This approach has recently been extended by introducing extensive rotamer sampling by means of which a better agreement with the experimental data can be achieved [15].

Molecular dynamics (MD) simulation is another powerful method for investigating the coupling of the ionisation equilibria and conformational flexibility. Different MD protocols and strategies have been employed to generate ensembles of structures to be used in p*K* calculations [13], [16]–[22]. Usually, the MD simulation is performed with a fixed protonation state of the protein, say, corresponding to a hypothetical pH at which all titratable groups are charged. The ionisation behaviour of the titratable groups, and their p*K* values, is predicted under the assumption that the conformational ensemble is pH independent. In this context, p*K* calculations based on conformational sampling extrapolate the properties of the protein along the pH scale in the same way as the calculations based on a single structure. To overcome this problem Baptista at al. [23] proposed MD combined with continuum electrostatic calculations in a way allowing the degree of protonation of the titratable groups to vary during the simulation. The calculated degree of protonation of the carboxyl groups of BPTI for constant pH 4 differed up to 0.15 in comparison with those computed from the crystal structure. No p*K* values have been reported. The methods for p*K* prediction based on constant pH MD simulation have been further developed by avoiding the introduction of partial protonation in the simulation protocol and by improving the conformational sampling methods [24]–[28].

In general, the computational approaches accounting for conformational flexibility reproduce the experimental values with an average error of about 0.6 pH units. The error for the sites buried in the protein interior is a bit higher, about 1.0 pH units [25], [29], [30]. Depending on the task, the prediction of the p*K* value of a given titratable group with an error even higher than the average could be considered as adequate [31]. In other cases, such as *in silico* screening of protein-ligand interactions, affinity design, etc. [29], the current accuracy may prove to be insufficient. Depending on the structural features of the protein molecule conformational changes in loop regions or cooperative motions occurring in the microsecond timescale can influence the ionisation equilibria [19]. A straightforward way to increase the accuracy of p*K* prediction is to extend the conformational space explored by MD simulation by increasing the length of the simulation, or by running multiple simulations. The prolongation of the simulation time can however not solve another key problem of the theoretical prediction of ionisation equilibria, namely the bias arising from the predetermined protonation state of the protein. This motivated us to look for an approach based on MD conformational sampling which addresses the above problem and the demands of a better prediction of the ionisation equilibria in proteins, yet keeping the computational costs to a moderate level.

We approach the problem of the protonation state bias with a multiple pH regime MD simulation, in which conformers are sampled from trajectories with different initial protonation state of the protein. In this way we extend the conformational space by exploring conformers expected to be populated at different pH intervals. For the purposes of the current investigation the 33 residue fragment of the GCN4 leucine zipper was chosen. Two useful features of this protein conditioned our choice. First, the experimental values for all titratable groups are available [32]. Second, since the protein is a homodimer, each titratable group in the protein is present in two copies, which are expected to have the same p*K* value due to the symmetry of the system. The difference between the p*K* values calculated for the corresponding pairs of titratable groups will then give a quantitative measure of the precision of the calculations.

In this paper we present the results of a total of 45 ns MD simulation carried out in explicit aqueous solution for three initial sets of initial protonation states of the protein: 1) neutral pH, with all titratable groups in their charged form; 2) acidic pH, with all glutamic acids in a protonated state; 3) basic pH, with all ε-amino groups in their deprotonated form. In all initial sets Tyr17 is in its protonated form. The quality of the results was assessed by comparison with calculations based on the X-ray structure of the protein and by comparison with the experimental data. The outcome of this study clearly fortifies the common opinion that p*K* calculations based solely on a single structure of the protein may give significant deviations from the experimental data even for sites that are not buried in the protein interior. We also demonstrate that the decisive factor for the improvement of the prediction is the variation of the initial protonation state of the protein.

## Methods

### Model building

The crystal structure of the GCN4 leucine zipper (Protein Data Bank entry code 2zta.pdb) was used to model the 33 residue fragment used in the experimental determination of the p*K* values [32]. In each chain of the molecule the N-terminal residue Arg1 was substituted by the dipeptide Gly0-Ser1, and Glu32 was appended to the C-terminus. All building manipulations were performed using CHARMM [33], [34] facilities and force field [35], [36]. The added amino acids were first subject to 100 steps Steepest Descent followed by 500 steps Adopted Basis Newton-Raphson (ABNR) minimisations. The rest of the protein atoms were restrained by a harmonic potential with a force constant of 300 kcal/mol/Å. The initial optimisation of the hydrogen atom positions was made by the same sequence of minimisations and harmonic constraints for the non-hydrogen atoms. The structure obtained in this way was used as initial structure in all MD simulations.

### Molecular dynamics simulation

The MD simulations were carried out using CHARMM programme [33], [34] with the Leapfrog Verlet integrator and the CHARMM22 all-atom force field [35], [36]. The protein molecule was centred in a rhombic dodecahedral box with lattice length of 69 Å and solvated with TIP3 water [37]. Appropriate amounts of NaCl and additional ions were added to keep the system neutrality and the ionic strength approximately 0.14 M for the different variants. This system was minimised as follows: First water molecules and all hydrogen atoms were minimised by Steepest Descent and ABNR, 100 steps each, keeping other atoms in a harmonic potential with force constant of 200 kcal/mol/Å. The procedure was repeated for the protein side chains whilst the force constant applied to the backbone atoms was reduced to 100 kcal/mol/Å. Finally, the above procedure was applied for all atoms without position restraints. The system was gradually heated from 48 K to 298 K within a 50 ps MD run and then equilibrated for 100 ps. Starting from this point, MD simulation of 15 ns was carried out with 2 fs integration step and periodic boundary conditions. Bond lengths involving hydrogen atoms were kept fixed by means of SHAKE [38]. All non-bonded interactions were smoothly shifted to zero at 12 Å cut-off. The non-bonded list was calculated out to 14 Å, and updated as soon as any atom had moved more than 1 Å. Three variants of protonation state of the protein were simulated with the above procedure (Table 1). Within the last 14 ns an average rmsd value of 2.3 Å was obtained for the backbone atoms compared to the initial structure.

**Table 1:** Charge content of the protein molecule used in the MD simulations.

Variant name	Protonation state
allC	All ionisable groups charged, tyrosine neutral
glu0	Glutamic acids neutral, all other charged, tyrosine neutral
lys0	Lysines neutral, all other charged, tyrosine neutral

### pK calculations

The degree of deprotonation of each individual ionisable site was calculated using the multiple hydrogen location approach [39]. This approach allows introduction of alternative position of the polar hydrogen atoms, such as histidine tautomers, rotamers of the hydroxyl groups, distinguished binding to the one or the other oxygen atoms in the carboxyl groups or distinguished dissociation of amino hydrogen atoms. In this approach a titratable group, *i*, is characterised by a given number of microscopic states corresponding to its protonation/deprotonation state and to the position of the hydrogen atom. The population of each microscopic state α is described by the statistical-mechanical formula
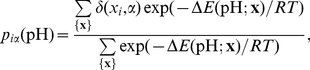
(1)where the summation is over the sets {**x**} of all protonation states of the protein. The sequence **x** = (*x*_1_, *x*_2_, …, *x_i_*, …, *x_N_*) contains *N* elements equal to the number of titratable groups. The elements *x_i_* enumerate the microscopic states of group *i*. The function *δ*(*x_i_*,α) is the Kronecker delta: *δ*(*x*,,α) = 1 if *x_i_* = α and *δ*(*x_i_*,α) = 0 if *x_i_*≠α, where α corresponds to a microscopic states enumerated by *x_i_*. The sequence **x** = (0, 0, …, 0) is chosen to be the reference state of the system. The term *ΔE*(**x**) is the change of the electrostatic energy from the reference state to state **x**:
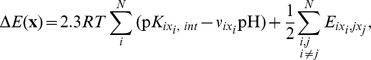
where indices *i* and *j* enumerate all sites and 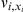 is the difference between the number of protons bound to site *i* in reference state and state *x_i_*. The electrostatic interaction energy between sites *i* and *j* in protonation state *x_i_* and *x_j_*, respectively, is given by 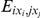. The term 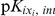
—being independent of the ionisation properties of the other groups in the protein—has a similar meaning as the intrinsic p*K* defined by Tanford [40]:
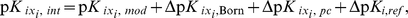
where 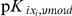 is the standard p*K* value of microscopic state *ix* of the *i-th* group [41]. The magnitude of 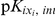 depends on the environment of the *i-th* group via the desolvation, 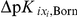, and the interactions with the permanent charges (non-titratable partial charges) in the protein, 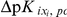. *Δ*p*K_i,ref_* accounts for the contribution of the charge-charge interactions between group *i* and all other titratable sites when they are in their reference protonation states. Hence, the magnitude of 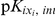 depends also on the choice of the protonation state of the titratable groups corresponding to the reference state of the system via *Δ*p*K_i,ref_*, making it dissimilar from Tanford's intrinsic p*K*. The choice of the reference state and the corresponding values of 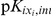 are those as in reference [41]. The outcome of the computations is the pH-dependencies of the populations, p_iα_, of the microscopic states of the individual titratable and polar groups. The p*K* value of a given titratable group is calculated from the pH-dependence of the population of its protonated state (for the imidazoles, amino and guanidine groups) or deprotonated state for (the carboxyl and the hydroxyl groups).

The terms comprising 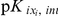 and 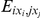 needed to obtain *ΔE*(**x**) are calculated by solving the linearised Poisson-Boltzmann equation using the finite difference method [42], [43] as described elsewhere [39], [41]. CHARMM parameter set 22 was used to assign partial charges, including those of the titratable functional groups. The van der Waals radii were taken from Rashin et al. [44]. The solvent probe radius and the thickness of the ion exclusion layer were set to 1.4 Å and 2.0 Å, respectively. Relative dielectric constants of 78 for the solvent and 4 for the protein moiety were used in the calculations. All calculations were made for ionic strength of 0.14 and 298 K.

### Coupling pK calculations with multiple pH MD simulation

Protein conformers (snapshot structures) were collected each 50 ps during the last 14 ns of the MD simulation. For each snapshot structure the populations of the microscopic states corresponding to the protonated species of the individual titratable groups were calculated using Eq. 1. In the terms of this equation this means that *p*_iα_(pH) is the population of the protonated form (α has a value set for the protonated species) of group *i* and given pH. The final populations of the protonated form of the individual groups are obtained by arithmetic averaging over the collected snapshot structures: 
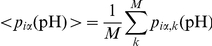
, where *M* is the number of snapshots. The midpoint of 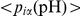
is then considered as the p*K* value of group *i* and designated as p*K*_1/2_.

To reduce the bias arising from the initially chosen protonation state of the protein three MD trajectories were generated with three different initial protonation states of the starting structure (Table 1). In this way, we expect to sample statistically relevant conformations corresponding to a wide pH range. Since in our approach there is no formal link between the individual sets of structures and pH we define it as follows: The trajectory generated with initial protonation state of the protein corresponding to all titratable groups in their charged forms (set allC, Table 1) is assumed to reflect the conformational flexibility of the molecule in the neutral pH region. Similarly, the sets glu0 and lys0 are expected to contain conformers with statistical relevance for the acidic and alkaline pH regions, respectively. Thus, instead of using several short MD simulations at different initial conditions but with the same protonation state of the protein, with the above approach we enhance the conformational space explored in a directed manner so that different pH regions are considered.

As pointed out above, there is no rigorous relation between pH and the conformational sets. Following the basic assumption of our approach, we expect that the different conformational sets appear with different weights in different pH regions. For instance, in the acidic pH region, where all carboxyl groups are protonated, the set glu0 should have weight of unity. With the increase of pH the population of glu0 decreases (the carboxyl groups deprotonate), whereas the population of allC (where the carboxyl groups are deprotonated) increases. The pH-dependence of the population of the individual sets of conformations is evaluated following the average deprotonation of the carboxyl groups calculated from set glu0, 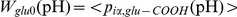, and of the amino groups calculated from set lys0, 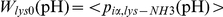. The angular brackets denote arithmetic average. The ionisation curves of the individual titratable groups, and consequently their p*K*_1/2_, are calculated by weighting the contributions of the three sets:
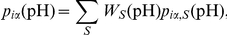
under the condition 
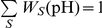
, where *S* enumerates the sets. The pH dependence of *W_S_* originates from the calculated ionisation equilibria within the same set, hence it is holding the same inaccuracy as the calculated ionisation curves themselves. This can be considered as a compromise by means of which the coupling of conformational flexibility at different pH and the cooperative ionisation behaviour of the whole multipole are taken into account in a simple way.

## Results and Discussion

The specific environment of the protein in the crystalline state, such as crystal contacts or ions with fixed position, may govern the molecule to adopt a conformation (or conformations) that is not necessarily dominantly populated in solution. This is an important issue in p*K* calculations since the ionisation behaviour of the individual titratable groups strongly depends on their local environment [45]. Thus for instance, changes in the desolvation energy arising from the conformational flexibility may introduce a p*K* shift of 2 pH units, or more, for groups which are not buried in the protein [7]. Hence, the identification of the most populated conformers of the molecule corresponding to certain conditions in solution, e.g. pH, is crucial for the theoretical prediction of the ionisation equilibria in proteins. Obviously, the wider the time window of the simulation, the larger is the conformational space explored. Also, with increased simulation time the bias arising from the initial structure is expected to decrease. However, the strategy of elongation of the simulation time alone is insufficient when pH dependent properties are of interest. The main reason is that for any length of MD trajectory only the conformers suitable for the initially chosen protonation state of the protein will be significantly populated. It will be illustrated below that the computational protocol, referred to as multiple pH regime, proposed in this work successfully addresses this problem giving a better agreement with the experimental observation in comparison to p*K* calculations based on either a single X-ray protein structure or on a set of structures sampled from a single MD trajectory (i.e. single protonation state of the protein).

### X-ray versus MD based pK calculations

**Figure 1:**
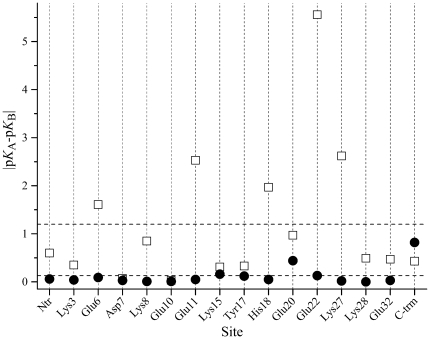
Difference between p*K*_1/2_ values calculated for chain A and chain B of GCN4 leucine zipper. Squares: calculations based on X-ray structure; Circles: calculations based on MD simulation. The upper and lower dashed lines correspond to |Δp*K*|*_av_* = 1.2 (X-ray) and |Δp*K*|*_av_* = 0.13 (MD), respectively.

**Figure 2:**
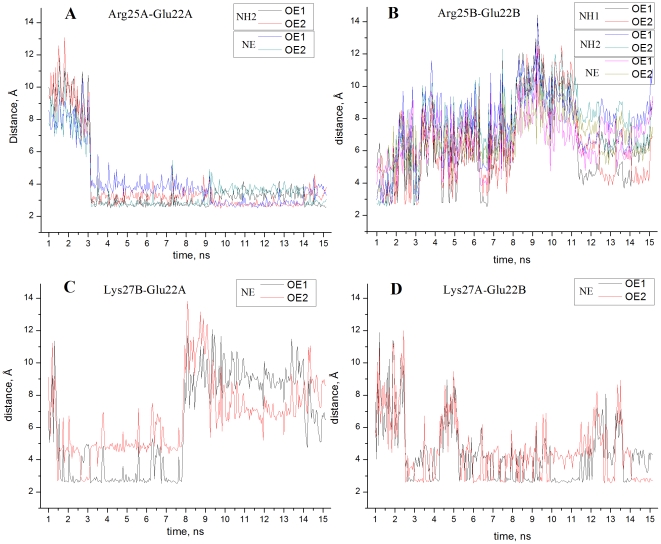
MD simulation (allC, **Table 1**) of salt bridge connectivity of Glu22. The distance between proton donors and proton acceptors of the functional groups forming salt bridges are plotted against time starting from 1 ns. The salt bridge partners are indicated in the panels. Phenomena clearly seen in the graphs, such as exchange of the hydrogen bond partners, are not discussed.

The computational data decidedly shows that p*K* calculations based on MD simulations improve the agreement with the experiment [13], [16]–[22]. Here we do not propose new evidence for that. We are rather interested in the extent of improvement that can be achieved by MD simulation with a moderate simulation time. A suitable feature of GCN4 leucine zipper is its homodimeric structure allowing the comparison of the ionisation of homologous pairs of titratable groups whose calculated p*K*_1/2_ are expected to be equal (see definition of p*K*_1/2_ in section “Computational Methods”). The difference between the p*K*_1/2_ values calculated for groups in a given pair, Δp*K_i_* = p*K*_1/2,*i*_(chain A)−p*K*_1/2,*i*_(chain B), can then be considered a measure for the precision of the computational protocol used in this study. This also is a useful—even though not sufficient—indicator for the completeness of the generated sets.

p*K*_1/2_ values obtained by the multiple pH MD simulations used in this work yield dramatically decreased |Δp*K*|*_i_* in comparison to that calculated on the basis of the X-ray structure (Fig. 1). Quantitatively this can be expressed by the average value of |Δp*K*|*_i_*, which decreases from 1.2 pH units in the case of single structure based calculations to 0.13 pH units after applying the MD protocol. The large difference in the p*K*_1/2_ values calculated for the single X-ray structure originates, as expected, from differences in the conformations and the environment of the homologous residues. Among them, Glu22 is characterised by a markedly large difference between the p*K*_1/2_ values in the two chains. The environment of the side chain of Glu22 has been subject of detailed experimental analysis [32]. NMR data [32] show that in solution Glu22 participates in two salt bridges: with Arg25 and with Lys27 from the other chain. The cross-link salt bridges Glu22A–Lys27B and Glu22B–Lys27A are observed in the X-ray structure. This is not the case for the salt bridge Glu22–Arg25, which is well defined (the distance between the proton donor and the proton acceptor is 2.83 Å) only in chain A. In chain B the functional groups of these residues are separated so that the distance between the atoms able to form a hydrogen bond varies between 6.06 and 6.45 Å. This difference between the charge configurations in the two chains causes the large divergence of the calculated p*K*_1/2_ values. As seen in Fig. 1, after applying the computational protocol the difference between the calculated p*K*_1/2_ decreases to 0.13 pH units. In agreement with the experimental observations [32] the salt bridge Glu22–Arg25 is present in the two chains (Fig. 2, panels A and B). However, in chain B this salt bridge is significantly less stable than that in chain A. An opposite symmetry is obtained for the cross-link salt bridges Glu22-Lys27 (panels C and D), where the salt bridge involving Glu22A is less populated. As a result, the p*K* shifts arising from the charge-charge interactions with the nearest environment and the salt bridge partners equalises, leading to a reduction of Δp*K_Glu22_*. In the set glu0—the one with competitive weight in the calculations of the ionisation curves of the glutamic acids—the above salt bridges are practically not present. This leads to further reduction of Δp*K_Glu22_*. The rearrangement of the configurations of the above clusters during the MD simulation also leads to equalization of the environments of Lys27 in the two chains and consequently to the reduction of Δp*K_Lys27_*.

**Figure 3:**
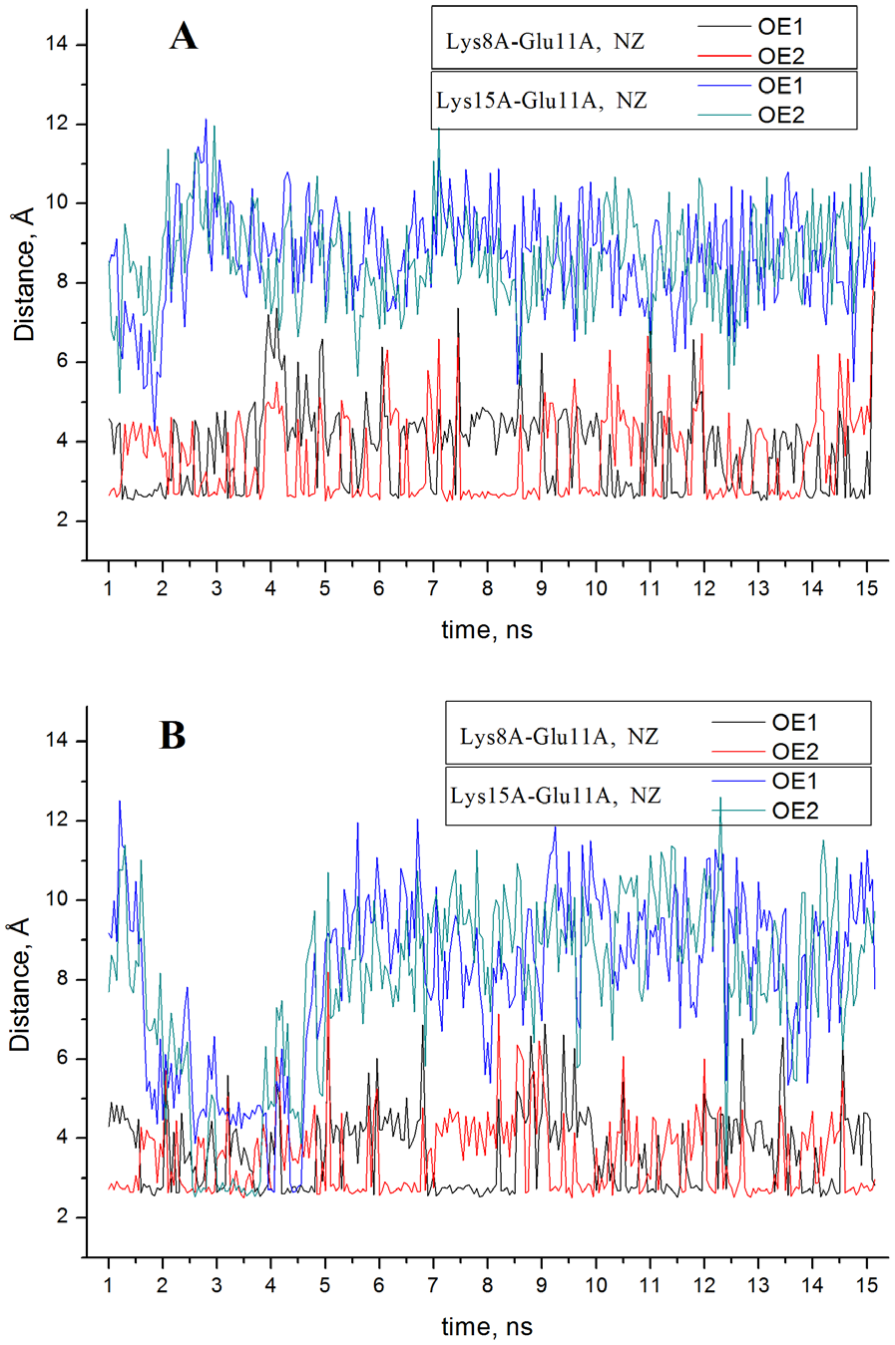
MD simulation (allC, **Table 1**) of salt bridge connectivity of Glu11. The salt bridge partners are indicated in the panels.

Very similar reasons for the large divergence of p*K*_1/2_ can be pointed out when considering the other titratable groups. Thus for instance, in the X-ray structure Glu11 forms a salt bridge with Lys8 in chain A, whereas in chain B this salt bridge is not observed. Also, in chain A Lys15 is in the nearest vicinity of Glu11 contributing to the stabilisation of the deprotonated form of this group. In chain B Lys15 is not in the environment of Glu11. The MD simulation reveals that the environments of Glu11 in the two chains become similar enough to diminish in this way Δp*K_Glu11_*. As seen in Fig. 3, the salt bridge Glu11-Lys8 is stable and present in both chains. The salt bridge Glu11-Lys15 is marginally present only in chain B. This result is in good agreement with the experimental evidence for the existence of the salt bridge Glu11-Lys8 and for the lack of the one between Glu11 and Lys15 [32].

In general, Fig. 1 illustrates that the MD simulation protocol used in this work significantly reduces the uncertainty compared p*K* calculations arising from the fixed protein structure. The average difference of the calculated p*K*_1/2_ values, |Δp*K*|*_av_* = 0.13, can be considered as a confidence interval for calculations based on conformational sampling. It should be noted that this small value may lead to the conclusion that MD simulation of 15 ns provides a set of structures unbiased from the initial structure. Although this is true in a majority of the cases, the example with Glu22 considered above shows that such a conclusion can be misleading. During 15 ns of simulation the side chain of Glu22 inhabits different environments in the two chains which are inherited from the initial structure. In chain A the salt bridge Glu22-Arg25 observed in the X-ray structure is well preserved during the MD simulation, whereas its counterpart in chani B remains unstable with a significantly lower lifetime (Fig. 2, panels A and B).

### Single versus multiple pH MD simulations, comparison with the experimental data

**Figure 4:**
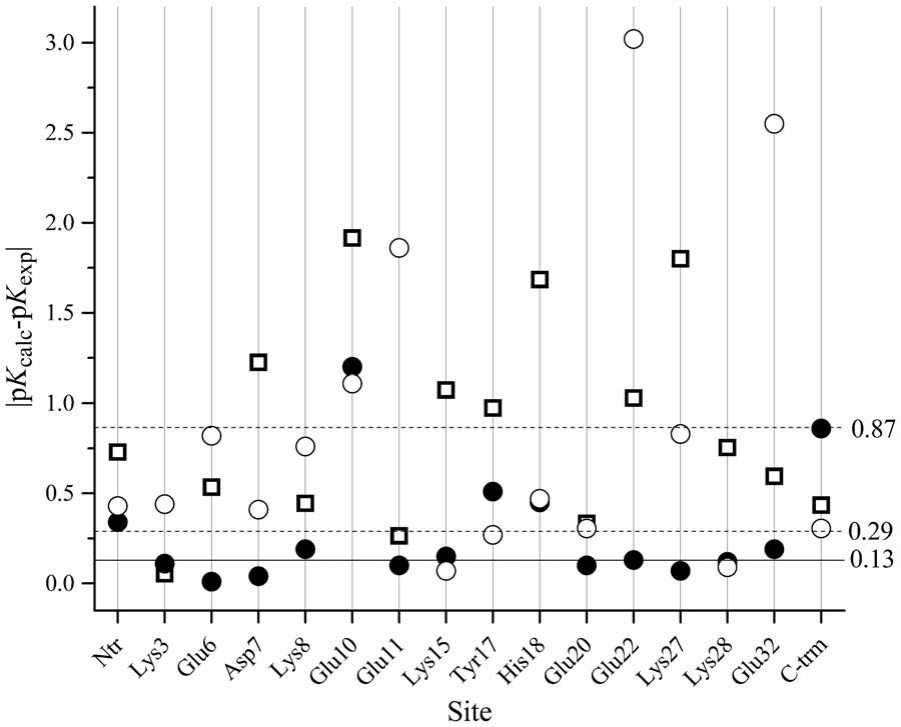
Comparison of the calculated p*K*_1/2_ values with the experimental data. Squares: calculations based on X-ray structure; Filled circles: calculations performed with weighted contribution of all sets; Open circles: calculations performed with the set of conformers when all titratable groups are charged (allC, Table 1). The horizontal dashed lines at 0.29 and 0.87 mark the mean deviation of the calculated p*K* values on the basis of MD simulation and X-ray single structure, respectively from the experimental p*K* values. The continuous horizontal line delimits the precision of the calculations (see also Fig. 1, Δp*K*|*_av_*).

The p*K*_1/2_ obtained as average of the values calculated from chains A and B of the GCN4 leucine zipper are compared with the experimental data of Matousek et al. [32] in Table 2. To facilitate comparison, the deviations of the calculated p*K*_1/2_ values from the experimental data are also plotted in Fig. 4. As seen, the multiple pH regime MD simulation considerably improves the agreement with the experimental data: the reproduction of the experimentally observed p*K* values is achieved with a mean deviation of 0.29 pH units versus 0.87 pH units in the case of p*K* calculations based on a single X-ray structure. This clearly shows that calculations based on a single X-ray structure are not enough for a reliable p*K* prediction. Also, this result shows that the conformers observed in the crystalline structure are not necessarily present with statistical significance in solution.

**Table 2:** Comparison of the p*K*_1/2_ values calculated in this work with the experimental values.

Site	p*K*_mod_	X-ray	allC onlya)	full MDb)	expc)
N-terminal	7.5	7.15	8.31	8.22	7.88
Lys3	10.4	10.73	11.22	10.89	10.78
Glu6	4.4	5.14	3.78	4.59	4.6
Asp7	4	4.70	3.89	3.52	3.48
Lys8	10.4	10.82	12.02	11.07	11.26
Glu10	4.4	5.86	5.05	5.14	3.94
Glu11	4.4	3.79	2.19	3.95	4.05
Lys15	10.4	11.70	10.55	10.47	10.62
Tyr17	9.6	10.80	10.09	10.39	9.82
His18	6.4	4.56	5.77	5.79	6.24
Glu20	4.4	4.72	4.08	4.48	4.38
Glu22	4.4	3.17	1.18	4.07	4.2
Lys27	10.4	9.3	11.93	11.03	11.1
Lys28	10.4	11.40	10.55	10.52	10.64
Glu32	4.4	4.02	2.07	4.43	4.62
C-terminal	3.8	4.46	3.725	3.17	4.03
**|p** ***K*** **_exp_−p** ***K*** **_calc_|** ***_av_***	**0.34**	**0.87**	**0.86**	**0.29**	
**rmsd**	**0.39**	**1.06**	**1.12**	**0.45**	

a) Calculation performed with the set of conformers when all titratable groups are charged (allC, Table 1).

b) Calculation performed with weighted contribution of all sets.

c) Experimental data taken from Matousek et al. [29].

The improvement of the agreement with the experimental data results from the introduction of sets of structures sampled with different protonation states of the initial structure. If only the set allC is used an improvement is achieved for about two thirds of the titratable groups. Consider Glu11 and Glu22 discussed above. The p*K*_1/2_ values of the two residues are shifted to low pH when calculated on the basis of the allC set only. As seen in Fig. 3, Glu11 participates in a salt bridge during the whole time window of structure sampling. As mentioned above, the observed behaviour of this residue is in agreement with the experimental data [32]. The contribution of the charge-charge interaction energy within the salt bridge Glu11-Lys8 to the p*K* shift of Glu11 is about 2.6 pH units on average reflected by the low p*K*_1/2_ calculated on the basis of the set allC. The situation of Glu22 is very similar. This residue forms salt bridges with Arg24 and Lys27, which are present during the whole simulated time interval. In spite of the asymmetric interactions involving Glu22 in the two chains of the molecule, the overall electrostatic influence of the closest vicinity appears to be the dominating factor for the dramatic shift of its p*K*_1/2_. In both cases the experimentally observed p*K* values are moderately shifted which contradicts the theoretical prediction based on the structure set allC. After introducing the contribution of the conformers in the set glu0, which in the pH range around 4 is equally populated to allC, a good agreement with the experimental data is achieved. Obviously, calculations based only on the allC set overestimate the contribution of the salt bridges.

The p*K* values measured for the GCN4 leucine zipper are quite similar to their standard values (all 16 values are within 1 pH unit of the standard value). Due to this the deviation of the calculated p*K* from the experimental values is comparable with that obtained by the Null model (using the standard p*K* values, p*K*_mod_, instead of calculating them), see Table 2. A narrow range of p*K* deviation from the standard value is in fact a feature of the majority of titratable groups in proteins. Thus for instance, in the proteins investigated by Aleksandrov et al. [30] the experimental p*K* values of only 1/4 of the titratable sites deviate more than 1 pH unit. For the sites with a small p*K* shift, |p*K*_exp_−p*K*_calc_|<1, the rms deviation obtained by these authors [30] (rmsd = 1.2) is similar to that of the Null model (rmsd = 1.1). The ultimate goal of any p*K* calculation is to predict the ionisation equilibria of both solvent accessible (presumably with small p*K* shifts) and buried (presumably with large p*K* shifts) titratable sites. In this context the Null model, having no predictive power, may only serve as a reference for a rough estimate of the accuracy of the theoretical approaches.

**Figure 5:**
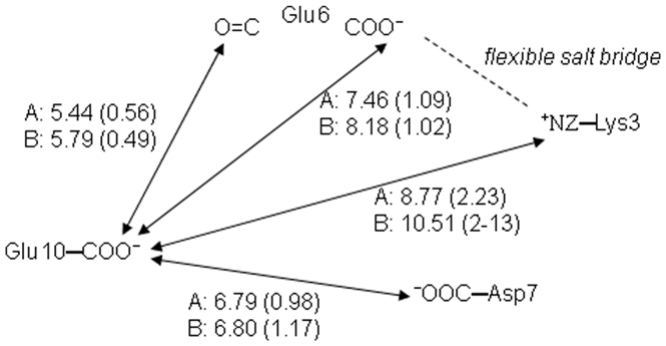
Schematic drawing of the environment of Glu11. The distances (Å) are averaged from the trajectory of the allC set. Letters A and B denote the corresponding chains of GCN4 leucine zipper. Values in parentheses are the standard deviations.

In our calculations two titratable sites (Glu10 and the C-terminal carboxyl group) do not follow the common trend of improvement of the p*K* prediction, and deviate more than 0.6 pH unit from the experimental value. Glu10 is a titratable site for which none of the discussed approaches gives agreement with the experimental data. This residue does not participate in salt bridges and as discussed by Matousek et al. [32] it inhabits an electrostatically unfavourable environment. The side chain of Glu10 is accessible to the solvent in the X-ray structure and remains such during the MD simulation. The average contributions of the desolvation and the interactions with the protein permanent charges is very small, 0.36 and −0.20 pH units, respectively, and practically compensate. Obviously, the up-shift calculated for this residue is due to like-charge repulsive Coulombic interactions, as suspected by Matousek et al. [32]. The nearest titratable groups around Glu10 are schematically drawn in Fig. 5. The configuration of charges in chain A gives about 1.1 kcal/mol destabilising energy for the charged carboxyl of Glu10. In chain B the charge configuration is practically the same, giving 1.2 kcal/mol unfavourable interaction energy. Although the up-shift is reduced by the MD simulation it remains 1.2 pH units. This discrepancy may arise from the peculiar configuration forming the environment of Glu10 which seems to be quasi-stable (Fig. 5): Glu6 forms a flexible salt bridge with Lys3 (∼50% occupation), whereas Asp7 is stabilised by Lys8, although they do not form a salt bridge. In the simulation with protonated carboxyl groups (set glu0) this configuration is stabilised because the unfavourable electrostatic interactions reduce for the neutral Glu10. One can presume that the time window of 15 ns is insufficient to break this configuration.

The C-terminal carboxyl group also belongs to the few groups for which the prediction of the experimental p*K* value failed. Judging from the slightly increased experimental p*K* value of this group in comparison with the standard value one can conclude that it does not participate in favourable electrostatic interactions. This assumption is partially confirmed by the simulation. In chain A, the C-terminal carboxyl group is not involved in charge-charge interactions with the neighbourhood, whereas at the same time the carboxyl group of the side chain of C-terminal residue (Glu32) forms a salt bridge with Lys27. In chain B, Lys27 forms a salt bridge with the C-terminal carboxyl instead of with the side chain. The occupancy of the salt bridges is 57% and 52%, respectively. As shown above (Fig. 2, C and D) Glu22 also competes for salt bridge formation with Lys27. The good agreement of the calculated p*K*_1/2_ for Lys27 with the experimental observation suggests that the electrostatic environment of this group is correctly simulated, including the salt bridges to the C-terminal residue. The discrepancy with the experimental data in the case of the C-terminal group can arise from the setup of the computational protocol. The protonation state of the protein used to sample the conformers for the set glu0 was defined so that only the glutamic acids are in their protonated forms. The C-terminal groups were however kept deprotonated. Therefore the salt bridge C-term–Lys27 found in chain B (allC set) remains well defined (with occupancy of 73%) in the glu0 set. This leads to overestimation of the calculated p*K*_1/2_ value of the C-terminal group. We did not create set of structures with protonated C-terminal group trying to keep the concept of variation of the protein protonation state formal and as simple as possible, i.e. altering the protonation state of the two types of groups most abundant in GCN4 leucine zipper and with standard p*K* values flanking the neutral pH region.

### Conclusions

The main outcome of this study is that extending the conformational space explored by MD simulation by generating sets with different initial protonation states of the protein molecule leads to a better agreement with the experimental data. In the context of the works on constant pH MD [24]–[28] discussed in the Introduction this is not an unexpected result. The benefit of this work is that the relatively good agreement with the experimental data is achieved by means of a simple approach, using a limited number of sets defined by formal criteria and moderate length of MD simulation. This makes the approach applicable for analysis of ionisation equilibria in proteins without employing large computational resources. Assuming independent motion of the side chains in the two helices of the protein, we have used the difference in the calculated p*K* for the homologous counterparts as a criterion for the precision of the method. Within 14 ns sampling of conformations this difference decreased to 0.13 pH units vs. 1.2 pH units obtained by calculations based on the X-ray structure. We accept this small value as satisfactory, thus we conclude that MD simulation of this size is appropriate for p*K* calculations. The approach failed to predict the ionisation equilibria of two groups with an accuracy less than or comparable with the commonly accepted one (0.6 pH units) [29]. We believe that in the two cases the failure originates from the limits set up for the calculations. In the case of the C-terminal group an expected overestimation of electrostatic interactions with the environment was obtained due to neglecting the possible conformational change of its deprotonated form. In the analysis of the dynamics of the environment of Glu10 we suspect that the simulation time is insufficient to allow the system to escape from a local energy minimum.
